# A look at Hypoactive Sexual Desire Disorder in Women

**DOI:** 10.1192/j.eurpsy.2025.634

**Published:** 2025-08-26

**Authors:** I. Santos, F. Cunha, R. Andrade, N. Castro, R. Cabral, C. Cunha, P. Pires, B. Melo

**Affiliations:** 1 Departamento de Psiquiatria e Saúde Mental, ULS Viseu Dão Lafões, Viseu, Portugal

## Abstract

**Introduction:**

Hypoactive sexual desire disorder (HSDD) is an underdiagnosed and poorly treated condition that is highly prevalent among women. Characterised by a persistent or recurrent deficiency of sexual desire, HSDD leads to significant personal distress and interpersonal difficulties, and can adversely affect emotional well-being and intimate relationships.

**Objectives:**

The aim of this review is to discuss the aetiology, diagnosis, and treatment of HSDD.

**Methods:**

A comprehensive literature search was conducted using the electronic database PubMed. The keywords used for the search included “Hypoactive Sexual Desire Disorder”, “treatment”, and “aetiology and diagnosis”.

**Results:**

The search yielded a total of five systematic reviews. These studies concluded that the aetiology of HSDD involves a complex interaction of biological, psychological, and sociocultural factors. The diagnosis of this disorder should include a comprehensive sexual and medical history to rule out other causes. Treatment options for HSDD are multifaceted, incorporating both pharmacological and non-pharmacological approaches.

**Conclusions:**

HSDD may be caused by biological factors such as a reduction in sexual excitation signals, an increase in sexual inhibition signals, or a combination of both. Testosterone plays a crucial role in initiating sexual activity, desire, and behaviour, through its influence on vaginal lubrication, sensation, and clitoral engorgement. Low oestrogen levels are associated with dyspareunia and changes in the vulvovaginal mucosa. Progesterone, serotonin, dopamine, and noradrenaline also play a role in the physiology of sexual desire. Psychological factors, particularly a lack of emotional intimacy, communication difficulties, negative body image perceptions and low self-esteem, can also reduce sexual desire. Depressive and anxiety disorders can significantly affect sexual desire. Sociocultural factors, such as religious beliefs and traditional values, can have a negative impact on sexuality. The diagnosis is made through a detailed clinical history, which may be supported by a screening tool, the Decreased Sexual Desire Screener (DSDS), as well as laboratory and imaging investigations. Identified modifiable factors, such as illicit substance abuse, sleep problems, medication use, and various medical and psychological factors, should be addressed first. For women without remaining modifiable factors who need psychological support, sex therapy, cognitive-behavioural therapy, and couples therapy are recommended. In premenopausal women, pharmacological treatment with flibanserin or bremelanotide may be considered. In postmenopausal women, hormonal therapy with testosterone may be considered off-label. The combination of psychological and pharmacological interventions is the most effective approach for HSDD. However, further studies are needed to better understand the pathological mechanisms of HSDD and to develop new therapeutic options.

**Disclosure of Interest:**

None Declared

